# Paediatric bone and soft tissue sarcoma

**DOI:** 10.1038/sj.bjc.6603635

**Published:** 2007-03-06

**Authors:** J Anderson

**Affiliations:** 1 Institute of Child Health and Great Ormond Street Hospital, London, UK

Paediatric bone and soft tissue sarcoma is a new text providing a comprehensive overview of paediatric sarcomas, largely with a North American perspective. Basic principles and recent concepts in the epidemiology, pathology, biology and clinical management are explored in 10 chapters. The text has 233 pages, 65 figures and 26 tables and is the effort of 27 North American contributors. The editor, Alberto Pappo, has recently joined the Texas Children's Cancer Centre as head of the solid tumour programme having worked previously in Toronto.

The first five chapters deal with general aspects of paediatric bone and soft tissue sarcomas with separate sections on epidemiology, pathological diagnosis, imaging, local control and drug discovery. Although slightly biased towards citing North American publications, the chapters generally provide a comprehensive overview of these important concepts and as such provide a good basic text for specialists or paediatric oncologists in training. Disappointingly, there is a lack of discussion of late effects, especially those resulting from radiotherapy. There is also lack of consideration of the important philosophical differences between North American and European treatment approaches, related to the decreased use of radiotherapy in Europe in soft tissue sarcoma management. Importantly, sections on molecular pathology and drug discovery are clearly written and easy for the general reader to understand. The imaging section is beautifully and comprehensively illustrated.

The next five chapters deal with distinct tumour types: rhabdomyosarcoma, non-rhabdomyosarcoma soft tissue sarcoma, fibrous tumours, Ewing family tumours and osteosarcoma. The rhabdomyosarcoma chapter is entitled ‘Biology and results of the North American Intergroup Rhabdomyosarcoma Trials’. It is to the editor's credit that this heading confirms the lack of reference to European trials and protocols. Whereas few in the field would disagree that the IRS trials have been at the forefront of rhabdomyosarcoma treatment advances, comparison with European approaches would be instructive here, especially as it marks an important area of controversy. The chapter on non-rhabdomyosarcoma soft tissue sarcomas has a more international flavour although space constraints and the number of different histologies under this single umbrella mean that discussions on optimal treatment approaches are a bit limited. This also reflects the lack of international collaborative trials for single histologies. There is a disappointing amount of repetition in this chapter.

The chapters on bone and fibrous tumours provide concise summaries of pathology, biology and therapy with comprehensive bibliographies. A large section on future developments in the Ewing's chapter is particularly welcome.

Paediatric sarcomas are rare, and the coupling of bone and soft tissue tumours is unusual. The book will be of value to all practitioners in paediatric oncology looking for some general reading to update themselves.

This volume deserves a place in the library of institutions involved in the care of children with malignant sarcomas.

